# Experience of 16 years and its associated challenges in the Field Epidemiology Training Program in Korea

**DOI:** 10.4178/epih.e2017058

**Published:** 2017-12-25

**Authors:** Moo-Sik Lee, Eun-Young Kim, Sang-Won Lee

**Affiliations:** 1 Department of Preventive Medicine, Konyang University College of Medicine, Daejeon, Korea; 2 Division of Public Health Preparedness and Response, Centers for Disease Control and Prevention, Cheongju, Korea

**Keywords:** Epidemiological investigations, Infectious diseases, Middle East Respiratory Syndrome, Korea

## Abstract

**OBJECTIVES:**

The field epidemiologist system of South Korea, which employs public health doctors who are relatively more readily available, was created in 1999 to ensure a ready supply of experts for epidemiological investigations and enable an effective response for new and reemerging infectious diseases. However, the 2015 outbreak of Middle East Respiratory Syndrome revealed limitations in the existing systems of management of field epidemiologists and communicable diseases.

**METHODS:**

The present study aims to evaluate data on current states, administrative reports, and other literature on the field epidemiologist system that has been in place in South Korea for 16 years since 1999 and to suggest appropriate future improvements in this system.

**RESULTS:**

By suggesting methods to evaluate the field epidemiologist system and training programs and by suggesting ways for the Korea Centers for Disease Control and Prevention to conduct evaluations on its own, the present study provides supporting evidence for improvement of systems for training of experts in epidemiological investigations. Moreover, based on the findings, this study also suggests methods to systematically train experts in communicable diseases management and a sustainable system to establish the basis of and develop strategies for a systematic and phased management of field epidemiologist training programs.

**CONCLUSIONS:**

The present study suggests the possibility of establishing dedicated training facilities, revising the guidelines on training and improvement of the competency of public health experts, while not limiting the scope of application to communicable diseases.

## INTRODUCTION

In the case of outbreaks of infectious diseases, it is imperative to find sources of infection through timely epidemiological investigations and to establish an appropriate prevention method based on the results of the investigations. However, inadequate initial epidemiological investigations sometimes lead to an inability to elucidate the causes of the spreading of infectious diseases and to establish clear disease prevention strategies as well as strategies to prevent recurrence. For timely prevention, it is necessary to find the sources and routes of infection through prompt epidemiological investigations in the initial stages of outbreak. To do this, the availability of epidemiology experts in infectious diseases and staff dedicated to infectious diseases is the most crucial factor [1]. Nevertheless, South Korea (hereafter Korea) currently lacks experienced field epidemiologists who have an adequate understanding of epidemiology of infectious diseases [2].

The expertise of field epidemiologists requires sufficient field data and an ability to make epidemiological judgments for making decisions relating to public health [3]. In the last Middle East Respiratory Syndrome (MERS) outbreak in May 2015 criticism on the lack of field epidemiologists available to conduct epidemiological investigations in emergency situations and the lack of continuity and expertise in epidemiological investigations gained support [4]. The Act on the Prevention and Management of Infectious Diseases, which aims to improve adequacy in the existing infectious disease management system revealed through the MERS outbreak, has been revised in part on July 6, 2015; the revisions include increases in the number and authority of field epidemiologists [5].

Therefore, the present paper aims to review the current Korean field epidemiologist system and training programs that seek to improve the competency of field epidemiologists.

## MATERIALS AND METHODS

This study aims to evaluate data on current states, administrative reports (included planning data, periodic and final reports, and evaluation reports of Korea field epidemiology training programs [KFETP], focus group interview and questionnaire survey), and other literature on the field epidemiologist management system that has been in place in Korea for 16 years since 1999, also seeks to suggest aspects that should be challenged in the future, and to suggest appropriate future improvements in this system. All tables were created from internal data of Korea Centers for Disease Control and Prevention (KCDC) and KFETP.

## RESULTS

### Introduction of Korean Field Epidemiology Training Programs (KFETP)

#### History of KFETP

The Chosun Rules on Committee for Infectious and Endemic Diseases established in 1920 (29th government general ordinance, July 14, 1920) included rules on the establishment and management of an epidemiological investigation bureau that functioned similarly to the present-day field epidemiologists [6-8]. The Law for the Prevention of Communicable Diseases, which was enacted as the 308th Law on February 2, 1954, established legal systems on Korea’s management of infectious diseases. The law was fully enforced when the enforced ordinance for the law was enacted on February 28, 1957 [6]. As part of the Research Project on the Training and Development of Experts in Infectious Diseases and Staff for Infectious Disease Management, which was established in 1999 to conduct research on precautions against newly emerging and reemerging infectious diseases, the field epidemiologist system was set in place. Initially, 19 public health practitioners were selected to receive 2 weeks of introductory education and were deployed at the Korea National Institute of Health (KNIH) and in each city and province; these epidemiologists were employed for 1 year for epidemiological investigations of infectious diseases. This was part of a pilot project, which employed public health practitioners who were relatively more available to effectively respond to new and reemerging infectious diseases by ensuring an available supply of experts in epidemiological investigations. In 2000, the Field Epidemiology Training Program (FETP), a training program focusing on field epidemiological investigations, was newly created. For the program, public health practitioners interested in epidemiology and infectious diseases were selected to become field epidemiologists who would be responsible for epidemiological investigations and surveillance of infectious diseases for at least 2 years. After 4 weeks of introductory training for field epidemiologists, the epidemiologists were deployed at the KNIH and in each city and province to conduct epidemiological investigations and surveillance of various infectious diseases [1].

On December 29, 2001, supporting evidence for national aid on the costs of training and education of field epidemiologists and on the investigation of adverse reactions to vaccines was prepared, and epidemiological investigations were subsequently systematized. Multiple revisions have been made since then, and the law was fully revised to the Law on Prevention and Management of Infectious Diseases to improve on problems in infectious disease management revealed during the 2009 outbreak of novel swineorigin influenza A (H1N1) [6]. The law was further improved in part on July 6, 2015, to improve on the issues found during the MERS outbreak in May 2015 [5].

#### Purpose of KFETP

The Centers for Disease Control and Prevention (CDC) defines FETP as an epidemiology and public health training and service program that establishes public health systems to improve sustainable public health competencies [9]. The KFETP has not been defined with ultimate aims as done by the CDC. However, the Rules on the Management of KCDC Field Epidemiologists states that the KFETP refers to a 2-year program, which includes introductory training for new field epidemiologists who will be appointed by the director of KCDC, mayors, or governors (less than 120 hr/ yr) and on-the-job training focusing on case studies provided to already appointed field epidemiologists (less than 50 hr/yr), according to Article 60 of the Law on Prevention and Management of Infectious Diseases [10]. Moreover, the purpose of FETP suggested in the 2015 project guidelines on the management of infectious diseases states that the FETP aims to enable field epidemiologists to conduct professional and active investigations in outbreaks by helping them acquire professional knowledge required for the improvement of field epidemiologist competencies and management of infectious diseases; to provide new knowledge and information on infectious diseases to field epidemiologists; and to improve expertise in epidemiological investigations through exchange of field experiences in epidemiological investigations [11].

### Current states, past experiences, and achievements of KFETP

#### Selection of field epidemiologists

Based on Article 26 of the enforced ordinance on the Law on Prevention and Management of Infectious Diseases, the Minister of Health and Welfare, mayors of each city, or governors of each province appoint field epidemiologists. The qualifications for field epidemiologists as specified by law include civil servants responsible for epidemiological investigations, disease prevention, or vaccination; public health practitioners employed according to the Special Law for Public Health in Rural Communities; medical practitioners as specified by Article 2 Clause 1 of the Medical Service Law; and other experts in fields pertaining to infectious diseases. Field epidemiologists are selected from those that qualify according to the above criteria [5]. Table 1 shows the specialties of field epidemiologists employed between 1999 and 2015 which were created from internal data of KCDC.

### Training program and contents

The KFETP, which is a 2-year-long program, consists of 1 introductory course (3 weeks), workshops (1/yr), on-the-job training (2/yr, 3 days each), and conferences for field epidemiologists (Table 2). Among public health practitioners selected and deployed as new field epidemiologists, licensed medical practitioners responsible for epidemiological investigations, civil servants responsible for disease prevention, epidemiological investigations, and vaccination, and experts in areas related to infectious diseases, those that wish to be trained are selected for the introductory course, which consists of a 3-week-long training on the acquisition of basic knowledge and techniques for epidemiology and infectious disease management. Workshops are conducted between the introductory course and on-the-job training and aim to form networks between newly appointed and experienced field epidemiologists and to promote discussions on the field epidemiologist system. The on-the-job trainings are conducted in September and March for central, municipal, and provincial field epidemiologists and for epidemiological investigation staff at quarantine centers and in each city and province. The trainings focus on epidemiological investigation cases to promote acquisition of expert knowledge in smaller projects and discussion of epidemiological investigation cases. Conferences for field epidemiologists are conducted separately during the Fall Conference for the Korean Society of Epidemiology owing to a memorandum of understanding (MOU signed with the society; these conferences aim to investigate cases of epidemiological investigation and to promote presentation and discussion of studies on the characteristics of infectious diseases [2,11].

### Courses for introductory and on-the-job training

The introductory training consists of the following 6 categories (Table 3): epidemiology and statistics of infectious diseases, waterborne and foodborne diseases, diseases for vaccination, responses to public health crisis, epidemiological investigations of other infectious diseases, and administrative measures. The on-the-job training consists of the 4 categories of methodologies of epidemiological research, epidemiology and statistics of infectious diseases, case discussion, and improvement of competency (Table 4). Courses in each category are determined through discussion of contracted educators and KCDC conducted before each training session [2].

### Areas of activities of field epidemiologists

Based on Article 26 Clause 2 of the enforced ordinance for the Law on the Prevention and Management of Infectious Diseases, the tasks of field epidemiologists are as follows: [12].

• Establishment of plans for epidemiological investigations
• Administration and analysis of epidemiological investigations
• Development of the criteria and methods of epidemiological investigations
• Training on techniques for epidemiological investigations
• Education and training on epidemiological investigations
• Epidemiological research on infectious diseases

Korean field epidemiologists play an important role in managing infectious diseases while conducting the tasks outlined by law as below: [13].

• In outbreaks or epidemics of infectious diseases, field epidemiologists conduct investigations in cooperation with the central government and municipal, county and district disease prevention agencies.
• In outbreaks or cases of eradicated infectious diseases, such as measles, field epidemiologists conduct case and epidemiological investigations.
• In outbreaks or cases of adverse reactions to vaccination, field epidemiologists conduct case and epidemiological investigations.
• When diseases that are not indigenous to Korea (e.g., *Plasmodium falciparum* malaria) are introduced from overseas, field epidemiologists conduct case investigations.

Moreover, field epidemiologists also play a significant role in national crises involving infectious diseases [13,14]: Investigation of measles epidemic and epidemiological investigation of adverse reactions to vaccination in 2000-2001; Case investigation of cholera and anthrax hoax terror in 2001; Case investigations during the severe acute respiratory syndrome epidemic in 2003; Case investigations of people that came into contact with AI during AI epidemics since 2003; Case and epidemic investigation during the H1N1 epidemic in 2009; Case and epidemic investigation during the MERS epidemic in 2015 (Table 5).

After retirement, field epidemiologists also act as educators in FETP, sharing information and their experiences with a sense of duty.

### Academic outcomes of field epidemiologists

Since 2010, training programs have been revised to improve the quality of education provided to field epidemiologists; as a result, field epidemiologists are now required to prepare and present at least 1 paper per year through conferences. The data collected through field epidemiologist activities are analyzed and orally presented at the conference held in the second year of the training program, and 6 to 8 academic research papers are published every year. However, academic activities are mostly conducted by central field epidemiologists, and not many field epidemiologists deployed at municipal or provincial levels are involved in academic activities.

### Mentorship program between field epidemiologists

In 2000, an advising professor system involving professors that lead field training from the start of FETP was planned, but the system was not managed properly [15]. For field epidemiologists working at the KCDC, multiple experts in related areas working in the same department can advise on performance of tasks and learning. However, since municipal and provincial field epidemiologists lack field experts who can provide training, they lack proper field training [2].

A one-on-one advising professor system for municipal and provincial field epidemiologists was implemented in 2014 upon signing of an MOU between the KCDC and the Korean Society of Epidemiology, which resulted in recommendations of professors from the Korean Society of Epidemiology. However, the system was conducted only in 2014, and no progress has been made since then. The system encouraged cities and provinces to voluntarily host epidemiological investigation task advisory meetings at least 3 times a year to enable field epidemiologists to directly receive advice on tasks from advising professors. The professors provided reviews and instructions on field epidemiological investigation results conducted by municipal and provincial epidemiologists; guidance on epidemiological investigation and research directions on nationally notifiable infectious diseases occurring in each city or province; and guidance on conference reports and publications of epidemiological investigation results [2,15].

### Evaluation of KFETP

KFETP are evaluated in terms of the management of training and training curricula. For the evaluation of the management of training, the KCDC and universities contracted for training hold meetings to evaluate each training phase [2]. When 1-year-long training programs are completed, a meeting for overall evaluation is hosted to provide feedback on the evaluation procedure and results such that the feedback can be reflected in the following year’s training plans. For evaluation of training curricula, trainees are evaluated through tests on their knowledge and practical skills acquired during the training. Moreover, they are surveyed on their satisfaction with the training program in terms of planning of training, operation of training, training media and environment, and training outcomes. The trainees also evaluate the curricula and lecturers [2].

### Results of evaluation of competency of field epidemiologists before and after introductory training

In 2015, an FETP competency evaluation was conducted before and after the introductory training for field epidemiologists. For the FETP competency evaluation, competency suggested by the CDC was translated into Korean and edited accordingly. Ten criteria and 16 items were evaluated. For techniques required for each competency, the trainees evaluated their own level of proficiency in the techniques on a 3-point scale [2]: point 1 refers to simply knowing the concept but lacking techniques to conduct tasks without others’ help; point 2 refers to knowing the concept and being able to conduct tasks with limited help from others; and point 3 refers to being able to conduct tasks without others’ help, to coach others, and to solve problems in a creative way.

According to evaluation conducted in 2015 before and after the introductory training for field epidemiologists, the scores improved significantly from 1.43 before the training to 2.04 after the training (p< 0.001). The descriptions of each area and competency are shown in Table 6.

## DISSCUSSION

We reviewed and summarized the current states and problems of the Korean field epidemiologist system and future improvements, discussed through focus group interviews (FGI) conducted with experts in preventive medicine and internal medicine in infectious diseases based on review of literature published within and outside of Korea; surveys conducted on experts, field epidemiologists, staff at KCDC, and municipal and provincial civil servants responsible for epidemiological investigation based on the FGI results; and workshops conducted with FETP and Epidemic Intelligence Service experts from CDC and Korean experts and researchers. In particular, the chapter focuses on ways to improve systems to train field epidemiologists; to improve programs for training, education, and evaluation of field epidemiologists; to establish and manage an institution for training; and to plan training of experts in epidemiological investigations from KCDC staff and municipal and provincial civil servants responsible for epidemiological investigation.

### Ways to improve systems to train field epidemiologists

The lack of continuity between expertise and experiences is recognized as the biggest problem in the current system. To recruit more field epidemiologists, it is important to promote occupational security (promotion and compensation), compensation at equal levels as private institutions, cooperation with various overseas institutions performing similar tasks, and field epidemiologists’ pride. In addition to compensation, promotion, and welfare, the following should also be implemented to recruit more field epidemiologists: a stable supply through establishment of public medical schools, connection to degree programs, provision of compensation at equal levels as private institutions while also providing high quality training and education, recruitment of experienced professors from private institutes to manage training and education to promote transfer of experienced experts through open positions, and improvement of expertise of field epidemiologists. The following opinions were also noted: exceptions from military service, transition from term system to continued service at related positions in the Ministry of Health and Welfare or KCDC, provision of incentives to field epidemiologists (current and retired), and management of long-term and short-term overseas training programs.

There is a need to improve the sense of duty and suggest future plans for field epidemiologists. First, the scope of work currently limited to infectious diseases should be expanded to chronic and environmental diseases, systematic support for preparation and response to national disasters should be provided, and the sense of duty should be promoted. Second, based on the excellent manpower, techniques, and experiences in Korea, communication with developed and third-world countries should be promoted to expand the scope of work and competency of field epidemiologists to outside of Korea. Third, beyond the term system, which is limited to KCDC, opportunities to work at the Ministry of Health and Welfare and other central government positions should be provided; at the same time, opportunities to work in related public service positions through promotion and transfer should be provided when field epidemiologists meet certain criteria.

It is also necessary to increase the financial compensation for field epidemiologists to a level equal to those at private institutions. Due to the intensifying delays in promotion, it is very difficult to unite civil servants through promotion. When promotion is limited, it is necessary to seek other methods to unite members through other methods of compensation. In such cases, provision of financial incentives is considered most effective; therefore, providing compensation at equal levels to private institutions should be considered. As another alternative, the experienced expert staff system, which was once available for Korean civil servants, can also be used when promotion is delayed due to an oversupply of medical civil servants with equal levels of experiences.

Opportunities for self-improvement should also be provided to field epidemiologists. Overseas training and participation in Korean and overseas conferences should be promoted to encourage continued self-development during employment. As in recruiting long-term employees in the army, medical school students should be recruited through scholarships, military service, degree programs, and preventive medical specialist programs.

Moreover, the current field epidemiologist system employing public health practitioners should also stay in place. Although field epidemiologists do not have to be doctors, epidemiological investigations of cases of infectious diseases require understanding of public health and clinical knowledge as they also involve patients. Thus, major activities and decision making mainly involve doctors. This is why it is difficult to recruit only public health experts in shortages of doctors applying to become field epidemiologists. It is also important to recruit adequate numbers of medical doctor field epidemiologists through allocation of a certain percentage of positions to doctors.

### Improvement of education, training, and evaluation programs for field epidemiologists.

It is necessary to set core competencies required for Korean field epidemiologists. According to the results of surveys on core competencies, it is necessary to set education and learning goals based on core competencies with consideration of the capabilities and scopes of capabilities required for field epidemiologists and the distribution of diseases in Korea.

Moreover, expert groups or committees to systematically support the field epidemiologist program should be recruited. Such groups or committees should be responsible for the maintenance of the overall quality of the program, development or revision of lectures or case studies in training curricula, and actual training and education of field epidemiologists. Further, an independent pool of dedicated staff or departments should be created to support programs within the KCDC.

Regarding training curricula, introductory and on-the-job training lasts for a total of 5 weeks, which is relatively shorter than FETP conducted overseas. It is necessary to expand the on-the-job training (2-3 days) rather than the introductory training. In particular, the on-the-job training should be expanded to conduct lectures, case studies, and practical training as in introductory training for each required competency. Moreover, since separate training on required core competencies was not provided for epidemiologists with different years of experience, education on core competency should be provided separately for epidemiologists with different experiences. Lectures should be prepared according to core competencies, and lecture time should be distributed accordingly for different core competencies. The time spent for case studies should be increased, and case studies should also be included in on-the-job training. Moreover, review and revision of questionnaires used in coursework training are also required.

In particular, seminars, which were not available previously, should be conducted every week. Since field epidemiologists are deployed at the KCDC and in each municipality and province, online video conferences (weekly) or in-person seminars (weekly or monthly) can be conducted to encourage the epidemiologists to report on their projects and receive guidance and feedback from experts.

For this, it is necessary to invite experts in various areas to conferences. Since some presentations do not involve discussions due to lack of time, this should be improved to enable presenters to receive in-depth guidance and feedback after presentation.

For field training, field managers should be present. The previous advising professor system cannot provide adequate guidance and feedback to trainees. Since experienced epidemiologists who can function as field managers at institutions at which epidemiologists are deployed are lacking, experienced epidemiologists should be encouraged to act as advisors even if they are not deployed at institutions. This would also improve mentorship. Technical advisors and supervisors are also required within institutions; in order for these people to acquire various in-field experiences, administrative support and dedicated staff are required.

For evaluation, criteria and requirement for completion should be made stricter. Moreover, shared parts on multisite FETP reports, completion on epidemiological investigations of epidemics, activities related to surveillance systems, and protocols for planned studies should be evaluated. For periodic evaluation of competency and project progress of field epidemiologists, on-the-job training can be expanded and conducted every 6 months. Evaluations should aim to enable epidemiologists to acquire competencies and complete projects. In other words, systems that can help epidemiologists to complete projects within 2 years should be prepared. Moreover, guidance and feedback are necessary for epidemiologists to complete projects with the help of stronger mentorship. Through the Epi-Aid (epidemiologic assistance) program, completion of projects (e.g., reports on epidemiological investigations of epidemics) should be encouraged.

### Plans for establishment of a dedicated training institution for selection and training of field epidemiologists

The current management of training for field epidemiologists have the following problems: limitations in continuity in education and training, lack of systematic investigation of demands for human resources, inadequate international networking systems, short-term systems for field epidemiologists, and limitations in ensuring a steady supply of human resources owing to field epidemiologists working in clinical areas rather than in public health areas after completion of service terms.

When the causes of the 2015 MERS epidemic were investigated, limitations in experts in field epidemiology, limitations in the current infectious disease management system, lack of field epidemiologists in infectious disease training and response training, and the need for organization and management of other public health human resources were emphasized.

To solve these problems, Korean and overseas experts, field epidemiologists, and researchers agreed that it would be most reasonable to operate a separate “training institute for field epidemiologists” with verified expertise, systematic management, continuous employment of human power (field epidemiologists), and excellence as a training institute.

To implement this in the long run, partial contracted training, which is currently being conducted by the KCDC and Konyang University College of Medicine, should be conducted in the near future. Dedicated staff within the KCDC capable of analyzing and managing such training institutes should then be recruited to form a committee for establishment of the training institute for field epidemiologists, also with the participation of various experts from within and outside of Korea, to train field epidemiologists in a practical manner.

The current Korean field epidemiologist system requires improvements in the system and training program evaluation. Moreover, the KCDC should also conduct its own evaluations to improve the systems to train experts in epidemiological investigations. Further, systematic methods for training experts in the management of infectious diseases, developing a basis for systematic and phased operation of FETP, and sustainable systems for development of strategies should be established. It is also necessary to revise the guidelines on training and improvement of the competency of experts in public health, and to establish dedicated training facilities.

## Figures and Tables

**Table 1.** Epidemic Intelligence Service officers’ specialties of Korea, 1999-2015

Location	Specialty	1999	2000	2001	2002	2003	2004	2005	2006	2007	2008	2009	2010	2011	2012	2013	2014	2015	Total
Central	Internal medicine	2	2	2	3	1	3								1			2	16
	Pediatrics	1			1	1		1	1	2	2	1	3		2	1	1		17
	Family medicine				1			2	1	2	1		1						8
	Preventive medicine		1											3					4
	Neurology							1			1		2		1	2	1	2	10
	Occupational and enviromental medicine									1			2			1	1		5
	Other specialty^1^	1	1	1											1	2			6
	Subtotal	4	4	3	5	2	3	4	2	5	4	1	8	3	5	6	3	4	66
Provincial	Internal medicine	4	4	3	15	9	10	11	9	6	5	7	4	2		2	4	4	99
	Pediatrics						1	1	2	2	2	1	4			2	1	1	17
	Family medicine					1			2	1	2			1					7
	Preventive medicine		1		1														2
	Neurology		1													1	2	1	5
	Occupational and environmental medicine															1			1
	Other specialty^2^	3	2		1	1		2		1	1	2		1	3	4	1	2	24
	General practitioner				1			1		1			3	2	4	1	1	1	15
	No information	7	7	4										1					19
	Subtotal	14	15	7	18	11	11	15	13	11	10	10	11	6	8	11	9	9	189
Total		18	19	10	23	13	14	19	15	16	14	11	19	9	13	17	12	13	255

Unit: persons.

1 Other specialty in central government: laboratory medicine, urology, obstetrics and gynecology, pathology etc.

2 Other specialty in provincial government: anesthesiology, urology, obstetrics and gynecology, thoracic surgery, radiology, orthopedics, rehabilitation medicine, surgery, ear, nose and throat, dermatology, ophthalmology, etc.

**Table 2.** Field epidemiology training program courses in Korea

Programs		Duration	Date	Contents
During the 1st year				
	Introductory course	3 wk	April - May	Acquisition of basic knowledge and techniques for epidemiology and infectious disease management
	Workshop	2 d	July	Discussion of improvements for the field epidemiologist system
	First on-the-job training	3 d	September	Sharing of experiences and knowledge gained through actual investigations and in-depth analysis and training on epidemiological investigations of infectious diseases
	Second on-the-job training	3 d	December	Sharing of experiences and knowledge gained through actual investigations and in-depth analysis and training on epidemiological investigations of infectious diseases
	Conference	1 d	February	Sharing of results and related information of epidemiological investigations
	Grand rounds	1/mo	Every month	Analysis of current outbreaks of infectious diseases and epidemiological investigation cases
During the 2nd year				
	Workshop	2 d	July	Discussion of improvements for the field epidemiologist system
	First on-the-job training	3 d	September	Sharing of experiences and knowledge gained through actual investigations and in-depth analysis and training on epidemiological investigations of infectious diseases
	Second on-the-job training	3 d	December	Sharing of experiences and knowledge gained through actual investigations and in-depth analysis and training on epidemiological investigations of infectious diseases
	Conference	1 d	February	Sharing of results and related information of epidemiological investigations
	Grand rounds	1/mo	Every month	Analysis of current outbreaks of infectious diseases and epidemiological investigation cases

**Table 3.** Courses and time spent for training during introductory education on field epidemiologists in 2015

Epidemiology and statistics of infectious diseases		Waterborne and foodborne diseases		Diseases for vaccination		Responses to public health crisis		Epidemiological investigation of other infectious diseases		Lecture on administrative measures	
Course	Time (hr)	Course	Time (hr)	Course	Time (hr)	Course	Time (hr)	Course	Time (hr)	Course	Time (hr)
Epidemiology of infectious diseases	3	Epidemiology of waterborne and foodborne diseases	1	Understanding national vaccination projects	1	Epidemiology of influenza	2	Understanding of insect vectors of diseases	2	Ice breaking	2
Methodologies in epidemiological research	3	Prevention and management of food poisoning and the role of the Ministry of Food and Drug Administration	1	Epidemiological investigation of infectious diseases for vaccination	1	Responses to human infections of Aland/VERS	1	Epidemiological investigations of mosquito-borne infectious diseases	1	Introduction of the field epidemiologist system	1
Epidemiological correlation and causality	2	Field epidemiological investigation of waterborne and foodborne diseases	2	Epidemiological investigation of adverse reactions to vaccination	1	Newest findings on new infectious diseases	2	Epidemiological investigation of febrile diseases common in fall	1	Public health and communication	2
Measurement of risk	1	Introduction on the collection of samples and laboratory diagnostic methods	2	Coursework training 3	4	Response system for biological terrorism	1	Epidemiology of healthcare-associated infectious diseases	2	Realities of the tasks of field epidemiologists	3
Investigation of spreading	2	Practice and tour of laboratory diagnostic methods	2			Responses to Al in Korea (H5N1 and H5N8)	1	Realities of epidemiological investigation of infections in hospitals	1	Field epidemiologists and public service after retirement	1
Measurement and screening test of diseases	2	Coursework training 1	4			Responses to and epidemiological investigation of Ebola occurrence in Korea	2	Epidemiological investigation of tuberculosis	2	Surveillance system of infectious diseases and management of information on infectious diseases	1
Completion of questionnaires and data entry	2	Coursework training 2	4			The role of rapid response bureau for prevention of human infection of Al and practice of putting g on and off of personal protective equipment (A, C, and D)	4	Zoonosis	2	Introduction of the Korean National Health and Nutrition Examination Surveys	1
Statistics for epidemiological investigation	2					Field responses to Ebola (activities of emergency disaster relief teams)	1	Non-nationally notifiable infectious diseases	1	Meeting with experienced field epidemiologists	4
How to use Excel and SPSS	3					Understanding epidemiological investigations seen in movies	2	Understanding high-risk pathogens and BSL3	2		
History of infectious diseases	2							Epidemiological investigation ofSFTS	1		
								Epidemiological investigation of respiratory diseases	2		
								Coursework training 4	4		
								Coursework training 5	4		
Total	22		16		7		16		28		15

AI, avian influenza; MERS, Middle East Respiratory Syndrome; BSL, bio-safety level; SFTS, severe fever with thrombocytopenia syndrome.

**Table 4.** Courses and time spent for training during on-the-job training for field epidemiologists in 2015

First on-the-job training			Second on-the-job training			Third on-the-job training		
Category	Course	Time (hr)	Category	Course	Time (hr)	Category	Course	Time (hr)
Epidemiology of infectious diseases	Case report of major epidemiological investigations of MERS outbreak: cases with uncertain routes of infection	1.0	Methodologies in epidemiological research	Revisions of pertussis guidelines	2.0	Methodologies in epidemiological research	Distribution of incubation period for infectious diseases and estimation and modeling of common exposure	2.5
	Case report of major epidemiological investigations of MERS outbreak: cases with long incubation periods	1.0	Epidemiology of infectious diseases	Management of latent tuberculosis	1.0		R statistics and graphs	2.5
	Public health practitioners and new infectious diseases	1.0	Discussion of cases	Review of MERS manual	2.0		Preparation of manuscripts in English for field epidemiologist	2.5
Discussion of cases	Discussion of future responses for sporadic occurrences of MERS	1.0		Review of the AI manual	2.0	Discussion of cases	Discussion for improvement of the field epidemiologist system	0.5
Improvement of competencies	Discussion of findings of MERS-related epidemiological investigations	2.0		Review of the Ebola manual	2.0	Improvement of competencies	EOC in Korea	0.5
				Discussion for improvement of the field epidemiologist system	5.0		Post-MERS measures from the Korean Medical Association	2.5
			Improvement of competencies	Revisions of pertussis guidelines	2.0		Doctors and KCDC seen through the media	2.5
							Field epidemiologist systems, such as EIS, FETP, and CSTE	1.0
							Evaluative tests for on-the-job training	0.5
Total		6.0			16.0			15.0

AI, avian influenza; EOC, Emergency Operations Center; EIS, Epidemic Intelligence Service; FETP, Field Epidemiology Training Program; CSTE, Council of State and Territorial Epidemiologists; MERS, Middle East Respiratory Syndrome; KCDC, Korea Centers for Disease Control and Prevention.

**Table 5.** Major outcomes of field epidemiologists by the year

Year	Disease	Region	Investigation (No. of patients)
2000	Malaria	National	Outbreak (32,647)
2001	Cholera	Goseong	Outbreak (18)
2003	Severe acute respiratory syndrome	National	Suspected case
2004	Hepatitis A virus	Gongju	Outbreak (105)
2005	Avian influenza virus	National	High risk group
2006	Shigellosis	Sancheong	Outbreak (198)
2007	Falciparum malaria	Busan	Confirmed case
	Human monkey pox	Seoul	Suspected case
2008	Norovirus	Churwon	Outbreak (625)
2009	Pandemic influenza A (H1N1)	National	Outbreak (>100,000)
	Hepatitis A virus	Seoul	Outbreak (33)
2010	Hepatitis A virus	Inje	Outbreak (44)
2011	Humidifier disinfectant lung injury	National	Outbreak (>100)
2012	Pertussis	Yeongam	Outbreak (154)
	Lyme disease	Hwacheon	Confirmed case
2015	Middle East Respiratory Syndrome	National	Outbreak (186)

**Table 6.** Evaluation results of competencies before and after field epidemiology training program in 2016

Description of each competency		n	Mean		Mean difference	Standard error of difference	p-value
			Before	After			
Epidemiological method							
	I can use epidemiological methods in conducting research to improve public health programs.	26	1.58	2.08	-0.50	0.71	0.001
	I can respond to epidemics of diseases	26	1.46	2.12	-0.66	0.69	<0.001
Statistics							
	I can analyze epidemiological data using appropriate statistical methods	26	1.54	2.08	-0.54	0.76	0.001
Public health surveillance							
	I can manage public health surveillance systems	26	1.46	2.08	-0.62	0.64	<0.001
Experiment and safety							
	I can use laboratory resources to support epidemiological activities	26	1.35	1.92	-0.57	0.81	0.001
Communication							
	I can develop documented public health communications	26	1.38	2.08	-0.70	0.68	<0.001
	I can develop and provide oral public health communications	26	1.46	2.08	-0.62	0.64	<0.001
Computer techniques							
	I can use computers for special tasks related to public health	26	1.62	2.04	-0.42	0.76	0.009
Management and leadership							
	I can manage field projects	26	1.35	2.04	-0.69	0.68	<0.001
	I can manage the staff and resources	26	1.50	2.04	-0.54	0.71	0.001
	I can become an effective team leader and member	26	1.46	2.04	-0.58	0.64	<0.001
	I can manage my own tasks	26	1.62	2.23	-0.61	0.64	<0.001
Preventive effects							
	I can apply simple tools to analyze economic efficiency	26	1.35	1.92	-0.57	0.70	<0.001
Teaching and mentoring							
	I can train public health experts	26	1.15	1.88	-0.73	0.67	<0.001
	I can mentor public health experts	26	1.19	2.00	-0.81	0.63	<0.001
Determination of the order of priority of diseases							
	I can evaluate the importance of diseases or national public health projects and determine the order of priority	26	1.42	1.96	-0.54	0.71	0.001
Total		26	1.43	2.04	-0.61	0.52	<0.001
