# Supplementary material for: Experience of 16 years and its associated challenges in the Field Epidemiology Training Program in Korea
Source: Epidemiol Health. 2017 Dec 25;39:e2017058. doi: 10.4178/epih.e2017058 (PMC5847970; doi:10.4178/epih.e2017058)
Supplement: Supplementary file 1 [file epih-39-e2017058-supplementary.pdf]

Volume: 39, Article ID: e2017058
https://doi.org/10.4178/epih.e2017058

Experience of 16 years and its associated challenges in the Field Epidemiology Training Program in Korea

Moo-Sik Lee, Eun-Young Kim, Sang-Won Lee*

Department of Preventive Medicine, College of Medicine, Konyang University
Division of Public Health Preparedness and Response, Centers for Disease Control and Prevention, Republic of Korea*

Corresponding author : Moo-Sik Lee

Department of Preventive Medicine, College of Medicine, Konyang University

158, Gwanjeodong-ro, Seo-gu, Daejeon 35365, Korea

E-mail: mslee@konyang.ac.kr

Volume: 39, Article ID: e2017058
https://doi.org/10.4178/epih.e2017058

Abstract

배경 : 한국의 역학조사관 제도는 1999 년 이는 신종 및 재출현 감염병에 대한 효과적인 대응을 위한 역학조사 전문 인력을 확보하기 위해 비교적 인력 수급이 용이한 공중보건의사 인력을 이용한 제도로 신설되어 시행되었다. 그러나 2015 년 중동호흡기증후군(메르스) 발생을 겪으면서 기존의 역학조사관 및 감염병 관리 제도의 운영상 한계점이 도출되었다.

연구목적 : 이 연구는 1999 년 이후 16 년간 한국에서 진행되어왔던 역학조사관 제도의 운영 현황자료, 각종 문헌, 행정보고서 등을 평가하고 바람직한 향후 역학조사관제도의 개선방향을 제시하고자 하였다.

결과 : 이 연구를 통하여 역학조사관 제도 및 교육프로그램의 평가 방법을 제시하고 향후 질병관리본부에서 자체적 평가의 방향을 제안함으로 역학조사 전문인력 양성을 위한 제도개선을 위한 근거 자료를 마련하였다. 또한 연구결과를 활용하여 보다 체계적인 감염병 관리 전문가 양성을 위한 방안과 역학조사관 교육 프로그램의 체계적인, 단계적인 운영 기반을 구축하고 전략 개발을 위한 지속가능한 수행체계를 제안하였다.

결론 : 감염병에만 국한되지 않고 공중보건 전문인력 양성 및 역량 강화의 가이드라인 개정 및 전문교육기관 설립에 대한 가능성을 제안하였다.

KEY WORDS: Epidemiological investigations, Infectious diseases, Middle East Respiratory Syndrome, Korea

*투고규정에 따라 영문원고는 국문원고 내의 표와 참고문헌 수와 상이합니다.

I. 서론

의학기술의 발달과 노인인구의 증가는 인간 집단의 주요 질병 패턴을 만성퇴행성 질환으로 변화시켰으며, 이에 따라 대부분의 보건학적 관심은 이 분야에 집중되어 왔다[1]. 하지만 최근 과거에 사라졌던 감염병이 재등장하고, 기후변화·세계화 등으로 새로운 감염병이 국내 출현하면서 감염성 질환이 다시 유행하는 양상을 보이고 있다[2]. 감염병 발생 시 신속한 역학조사를 실시하여 조기에 감염원을 파악하고 이를 바탕으로 한 적절한 방역관리가 중요하게 요구되고 있으나, 초기 역학조사의 미비로 감염병 유행의 원인을 규명하지 못하고 명확한 방역조치 및 재발 방지 대책을 세울 수 없는 사례가 발생하기도 한다. 신속한 방역조치를 위해서는 감염병 발생 초기에 신속한 역학조사를 수행하여 감염원 및 감염경로 등을 파악하는 것이 무엇보다도 중요하며, 이를 위해서는 감염병 역학전문가 및 감염병 전담요원 등 인적요소가 가장 중요한 요소라고 할 수 있다[3]. 그러나 아쉽게도 현재 국내에서 현장 경험이 풍부하면서 감염병 역학에 대한 이해를 하고 있는 현장 역학전문가는 부족한 실정이다[4].

현장 역학전문가의 전문성은 현장의 데이타를 충분히 가지고 공중보건 의사결정을 위한 역학적 판단 능력이 있어야 한다[5]. 지난 5 월 중동호흡기증후군(메르스) 발생으로 인한 역학조사과정에서 이를 담당할 수 있는 국내 현장 역학전문가의 수는 긴급한 상황에 대처하기에는 턱없이 부족하다는 비판과 역학조사 업무 종사의 연속성과 전문성이 떨어진다는 비판을 받았다[6]. 중동호흡기증후군(메르스) 발생 이후 기존 감염병 관리 제도의 운영에서 나타난 각종 미비점들을 개선·보완하려는 취지의 “감염병의 예방 및 관리에 관한 법률”이 지난 2015 년 7 월 6 일 일부 개정되었으며, 그 내용 중에는 역학조사관 수의 증가와 권한을 강화하는 내용이 신설되었다[7].

이 글은 2015 년 중동호흡기증후군(메르스) 발생 이후 감염병 관리에 있어서 법령 및 제도의 개선이 요구되고 있는 시점에서 그간의 우리나라 역학조사관 제도와 역학조사관의 역량 강화를 위한 교육 프로그램을 검토하고, 앞으로 도전해나가야 할 과제들을 제안해보고자 하였다.

II. 한국의 역학조사관 교육 프로그램의 소개

한국의 역학조사관 교육 프로그램의 역사

1920 년 ‘조선전염병및지방병조사위원회규정’(총독부령 제 29 호, 1920. 7. 14.)은 현재와 유사한 역학조사반의 기능과 역할을 위한 설치·운영규정을 두고 있었다[8-10]. 이후 1954 년 2 월 2 일 법률 제 308 호로 제정된 ‘전염병예방법’에서 한국의 감염병 관리에 관한 법적 체계를 마련하게

되었고, 본격적으로 시행된 것은 1957 년 2 월 28 일 전염병예방법 시행령이 제정된 시점이라고 볼 수 있다[8]. 역학조사관 제도는 1999 년 신종, 재출현 전염병 대비 정책연구로 "전염병 전문가 양성 및 전염병 관리요원의 개발 연구사업"의 일환으로 공중보건의사 중에서 19 명을 선발하여 2 주간의 기본교육 후 국립보건원 및 각 시·도에 배치하고 1 년 동안 감염병 유행 역학조사에 역학전문가로 활약하도록 하였다. 이는 신종 및 재출현 감염병에 대한 효과적인 대응을 위한 역학조사 전문 인력을 확보하기 위해 비교적 인력 수급이 용이한 공중보건의사 인력을 이용한 시범사업의 일환이었다. 2000 년부터는 현장 역학조사 중심의 교육훈련 과정인 역학조사 전문가 과정(field epidemiology training program, 이하 FETP)를 신설하여 공중보건의사 중에서 역학 및 감염병에 관심이 있고, 2 년 이상 감염병 역학조사 및 감시체계 업무를 담당할 지원자를 대상으로 '역학조사관'을 선발하여, 약 4 주간의 역학조사관 기본교육 과정을 거친 후 국립보건원 및 시·도에서 각종 감염병의 유행 역학조사 및 감시체계와 관련된 활동을 하도록 하였다[3].

2000 년 1 월 12 일 '전염병예방법이'이 개정되면서 역학조사에 관한 근거규정이 마련되었고, 2001 년 12 월 29 일 '예방접종이상반응'에 관한 조사 및 역학조사관 교육·훈련에 대한 경비의 국고보조의 근거가 마련되면서 역학조사에 대한 제도가 체계화되기 시작하였다. 이후 여러 번의 개정이 있었지만, 2009 년 신종인플루엔자 대유행 이후 감염병 관리 제도의 미비점을 보완하기 위해 '감염병의예방및관리에관한법률'로 전면개정하였고[8], 2015 년 5 월 중동호흡기증후군(메르스) 발생 이후 나타난 미비점들을 개선하기 위해 2015 년 7 월 6 일 일부 개정이 되었다[7].

한국의 역학조사관 교육 프로그램의 목적 및 목표

미국질병통제예방센터(centers for disease control and prevention, 이하 CDC)는 FETP 를 '지속 가능한 보건 역량 강화를 위하여 공중 보건 시스템의 능력을 구축하고 적용 가능한 역학 및 공중보건의 교육 및 서비스 프로그램이다'라고 정의하고 있다[11]. 한국의 역학조사관 교육 프로그램을 CDC 와 같이 궁극적 목적을 포함하여 간략하게 정의하지는 않았지만, 2015 년에 적용된 '질병관리본부 역학조사반 운영 등에 관한 규정'을 살펴보면 "역학조사관 교육과정(Korea Field Epidemiology Training Program(이하 'KFETP'라 한다.)은 「감염병의 예방 및 관리에 관한 법률」 제 60 조에 의하여 질병관리본부장 또는 시·도지사가 임명 예정인 역학조사관을 대상으로 실시하는 신규자 기본교육(연간 120 시간 이내)과 임명된 역학조사관을 대상으로 실시하는 사례중심의 지속교육(연간 50 시간 이내)으로 2 년 과정을 말한다"라고 정의하고 있다[12]. 또한, 2015 년 감염병 관리 사업지침에 제시된 역학조사관 교육 프로그램의 목적을 살펴보면 "역학조사관의 업무수행능력 향상 및 감염병 관리에 필요한 전문지식을 습득하게 하여 감염병 발생 시 전문적이고 능동적인 역학조사업무를 수행하며, 역학조사관에게 감염병과 관련된 새로운 지식과 정보를

Volume: 39, Article ID: e2017058
https://doi.org/10.4178/epih.e2017058

제공하고, 역학조사 현장 경험의 교류를 통한 역학조사업무의 전문성을 제고하고자 한다"라고 기술하고 있다[2].

역학조사관 교육 프로그램을 위탁운영하고 있는 대학에서 제안한 한국 역학조사관 교육 프로그램의 목표는 다음과 같다.

. 문제해결능력 및 전문성을 갖춘 감염병 역학조사관 육성

. 지역사회 감염병 예방·관리에 지도력을 갖춘 감염병 역학조사관 육성

III. 한국의 역학조사관 교육 프로그램 현황 및 그간 경험과 성과

역학조사관의 선발

역학조사관의 선발은 감염병의 예방 및 관리에 관한 법률 시행령 제 26 조를 근거하여 보건복지부장관 및 시・도지사가 역학조사관을 임명한다. 법에 근거한 역학조사관의 자격은 방역, 역학조사 또는 예방접종 업무를 담당하는 공무원,「농어촌 등 보건의료를 위한 특별조치법」에 따라 채용된 공중보건의사, 「의료법」 제 2 조제 1 항에 따른 의료인, 그 밖에 감염병 등과 관련된 분야의 전문가 중 선발하여 임명된다[7]. 1999 년에서 2012 년기간 동안의 역학조사관의 전공별 분포는 다음과 같다(Table 1).

Table 1. EIS* officers' specialties of Korea, 1999-2015

unit: persons

Location	Specialty	'99	'00	'01	'02	'03	'04	'05	'06	'07	'08	'09	'10	'11	'12	'13	'14	'15	total
Central	Internal medicine	2	2	2	3	1	3								1			2	16
	Pediatrics	1			1	1		1	1	2	2	1	3		2	1	1		17
	Family medicine				1			2	1	2	1		1						8
	Preventive medicine		1											3					4
	Neurology							1			1		2		1	2	1	2	10
	Occupational and Environmental medicine									1			2			1	1		5
	Other specialty**	1	1	1											1	2			6
	Subtotal	4	4	3	5	2	3	4	2	5	4	1	8	3	5	6	3	4	66
Provincial	Internal medicine	4	4	3	15	9	10	11	9	6	5	7	4	2		2	4	4	99
	Pediatrics						1	1	2	2	2	1	4			2	1	1	17
	Family medicine					1			2	1	2			1					7
	Preventive medicine		1		1														2
	Neurology		1													1	2	1	5
	Occupational and Environmental															1			1

Volume: 39, Article ID: e2017058
https://doi.org/10.4178/epih.e2017058

medicine																		
Other specialty**	3	2		1	1		2		1	1	2		1	3	4	1	2	24
General practitioner				1			1		1			3	2	4	1	1	1	15
No information	7	7	4										1					19
Subtotal	14	15	7	18	11	11	15	13	11	10	10	11	6	8	11	9	9	189
Total	18	19	10	23	13	14	19	15	16	14	11	19	9	13	17	12	13	255

* Epidemic Intelligence Service

** Other specialty in central government: laboratory medicine, urology, obstetrics and gynecology, pathology etc. ; +Other specialty in provincial government: anesthesiology, urology, obstetrics and gynecology, thoracic surgery, radiology, orthopedics, rehabilitation medicine, surgery, ear, nose and throat, dermatology, ophthalmology, etc.

교육과정과 내용

한국의 역학조사관 교육 프로그램의 구성은 기본교육과정(introductory course) 1 회(3 주), 워크샵(연 1 회), 지속교육과정(on-the-Job Training) 연 2 회(2 박 3 일), 역학조사관 학술대회로 구성되어 있으며, 2 년 과정이다(Table 2). 기본교육과정은 신규 역학조사관으로 선발·배치된 공중보건의사 또는 의료인 면허소지 역학조사 업무 수행자, 방역·역학조사·예방접종 담당 공무원, 감염병 등 관련분야 전문가 중 교육 희망자를 선발하여 3 주 동안 역학 및 감염병 관리에 대한 기본 지식과 기술 습득을 위한 교육을 진행한다. 워크샵은 기본교육과정과 지속교육과정 사이에 개최되며, 신규로 임명된 역학조사관과 기존의 역학조사관의 네트워크 형성과 역학조사관 제도 등에 대한 논의가 이루어진다. 지속교육과정은 9 월과 3 월에 실시되며, 중앙 및 시·도 역학조사관, 검역소, 시·도 소속 역학조사요원을 대상으로 역학조사 사례를 중심으로 세부과제별 전문지식 학습, 역학조사 활동 사례 토의 등을 진행한다. 역학조사관 학술대회는 한국역학회와 MOU 를 체결로 한국역학회 추계학술대회 내 별도 세션으로 운영하고 있으며, 역학조사 사례조사 및 감염병 특성 연구 발표 및 토의를 진행하게 된다[2, 4].

2000 년 역학조사관 교육과정이 처음 개설운영된 후 2014 년도까지 역학조사관 교육과정은 2 년의 과정으로 구성하여, 초기 1 년차에는 기본교육과 1 차 워크샵, 1 차 지속교육과 2 차 지속교육, 학술대회를 진행하였고, 2 년차에는 2 차 워크샵과 3 차 지속교육, 4 차 지속교육 그리고 학술대회를 진행하였다. 2015 년 워크샵으로 진행되었던 교육과정이 1 차 지속교육으로 명칭을 변경하고, 과거의 워크샵 목적 및 성격을 유지하면서 교육을 좀 더 강화하는 내용으로 발전하였다. 따라서, 2015 년 교육과정부터는 2 년 과정은 동일하나, 1 년차에는 기본교육과 1 차 지속교육, 2 차 지속교육, 3 차 지속교육, 학술대회, 2 년차에 4 차 지속교육, 5 차 지속교육, 6 차 지속교육 그리고 학술대회로 운영되고 있다. 즉, 총 2 년 과정에서 기본교육 1 회와 지속교육 6 회, 그리고 학술대회 발표를 거쳐야 역학조사관 교육과정을 모두 이수하게 되는 것이다.

Volume: 39, Article ID: e2017058
https://doi.org/10.4178/epih.e2017058

기본교육과정에서의 주 내용은 역학 및 감염병 관리에 대한 기본지식과 기술을 습득이며, 역학조사관 선발 후 3 주간 진행되었다. 보통 2 분기에 시작되었으며, 4 월 중순에서 5 월 초까지 진행하였다. 워크샵(2015 년 이후 1 차 지속교육으로 명칭 변경)은 역학조사관 제도 개선사항을 위한 토론 및 화합을 위한 것으로 7 월 중에 개최되었다. 지속교육은 활동사례를 통한 경험, 지식 공유 및 감염병 역학조사 심층분석과 심화교육이 진행되었으며, 연 2 회 각 3 일간 진행되었다. 역학조사관 학술대회는 연 1 회 실시하고 있으며, 2 년차 교육과정의 교육생들의 연좌로 준비되었다. 질병관리본부는 한국역학회와 MOU 를 체결하고, 역학조사관 학술대회를 한국역학회 추계학술대회 내 세션으로 운영하였으며, 학술대회 개최 시기는 주로 11 월 또는 12 월에 개최하였다. 학술대회에서는 주로 역학조사 사례조사 및 감염병 특성 연구를 발표하고, 전문가 토의를 진행하였다[2, 4].

Table 2. Field epidemiology training program courses in Korea.

	기간	시행일	주요 내용
1년차			
기본교육	3주	4~5월	역학 및 감염병 관리에 대한 기본 지식과 기술 습득

워크샵	2일	7월	역학조사관 제도 개선사항을 위한 토론 및 화합
1차 지속교육	3일	9월	활동사례를 통한 경험, 지식 공유 및 감염병 역학조사 심층분석 및 심화교육
2차 지속교육	3일	12월	활동사례를 통한 경험, 지식 공유 및 감염병 역학조사 심층분석 및 심화교육
학술대회	1일	2월	역학조사 결과 및 관련 정보 공유
Grand Rounds	1회/월	매월	감염병 발생 현황 및 역학조사 사례 분석
2년차			
워크샵	2일	7월	역학조사관 제도 개선사항을 위한 토론 및 화합
1차 지속교육	3일	9월	활동사례를 통한 경험, 지식 공유 및 감염병 역학조사 심층분석 및 심화교육
2차 지속교육	3일	12월	활동사례를 통한 경험, 지식 공유 및 감염병 역학조사 심층분석 및 심화교육
학술대회	1일	2월	역학조사 결과 및 관련 정보 공유
Grand Rounds	1회/월	매월	감염병 발생 현황 및 역학조사 사례 분석

기본교육과 지속교육 교과목

기본교육과정은 크게 감염병 역학 및 통계, 수인성·식품매개질환, 예방접종대상질환, 공중보건위기대응, 그 외 감염병 역학조사, 행정 관련 강의 등 6 가지 카테고리로(Table 3), 지속교육과정은 크게 역학연구방법론, 감염병 역학 및 통계, 사례토의, 역량강화로 4 가지 카테고리로 나누어져 있으며(Table 3), 그 세부 교과목에 대해서는 매 교육 시행 전 위탁교육전문가와 질병관리본부에서 논의를 거쳐 결정하게 된다[4].

Table 3. 2015년 역학조사관 기본교육과정의 교과목 구성 및 교육 시간

감염병 역학 및 통계		수인성·식품매개질환		예방접종대상질환		공중보건위기대응		그 외 감염병 역학조사		행정 관련 강의 등	
교과목	시간	교과목	시간	교과목	시간	교과목	시간	교과목	시간	교과목	시간
감염병 역학	3	수인성·식품매개 질환의 역학	1	국가 예방 접종사업의 이해	1	인플루에자의 역학	2	질병매개곤충의 이해	2	ICE Breaking	2
역학연구 방법론	3	식중독 예방·관리와 식약처의 역할	1	예방접종대상 감염병 역학조사	1	AI 인체감염 및 MERS 대응	1	모기매개 감염병 역학조사	1	역학조사관 제도 소개	1
역학적 연관성과	2	수인성·식품매개질환의 현장	2	예방접종 후 이상반응	1	신종 감염병의 최신지견	2	가을철 발열성질환	1	보건과 소통	2

Volume: 39, Article ID: e2017058
https://doi.org/10.4178/epih.e2017058

인과관계		역학조사		역학조사				역학조사			
위험도의 측정	1	검체 채취와 실험실 진단법 총론	2	Coursework Training 3	4	생물테러 대응체계	1	의료관련 감염병의 역학	2	역학조사관업무의 실제	3
유행조사	2	실험실 진단법 실습 및 견학	2			국내 발생 AI(H5N1, H5N8) 대응	1	병원감염역학조사의 실제	1	역학조사관 그리고 이후의 공직생활	1
질병수준의 측정과 선별검사	2	Coursework Training 1	4			국내 에볼라 대응 및 역학조사	2	결핵의 역학조사	2	감염병 감시체계와 감염병 정보관리	1
설문지 작성과 자료입력	2	Coursework Training 2	4			AI 인체감염 예방을 위한신속대응반 의역할및개인보호구(A,C,D)착·탈의 실습	4	인수공통감염병	2	국민건강영양조사사업 소개	1
역학조사를 위한 통계학	2					에볼라현장 대응(긴급구호대 활동)	1	비법정감염병 역학조사	1	선배역학 조사관과의 만남	4
엑셀 및 SPSS 사용법	3					영화속의 역학조사 이해하기	2	고위험병원체와 BSL3 이해	2		
감염병의 역사	2							SFTS 역학조사	1		
								호흡기질환 역학조사	2		
								Coursework Training 4	4		
								Coursework Training 5	4		
계	22	계	16	계	7	계	16	계	28	계	15

Table 4. 2015년 역학조사관 지속교육과정의 교과목 구성 및 교육시간

1차 지속교육			2차 지속교육			3차 지속교육		
구분	교과목	시간	구분	교과목	시간	구분	교과목	시간
감염병 역학	메르스 유행의 주요 역학조사 사례 보고 : 감염경로 불분명 사례	1.0	역학연구 방법론	백일해 지침 개정 안내	2.0	역학연구 방법론	감염병의 잠복기분포와 공동노출일 추정 및 모델링	2.5
	메르스 유행의 주요 역학조사 사례 보고 : 잠복기가 긴 사례	1.0	감염병 역학	잠복결핵 관리	1.0		R 통계와 그래프	2.5
	공중보건의사와 신종감염병	1.0	사례토의	메르스 메뉴얼 검토	2.0		역학조사관을 위한 영어 논문 작성법	2.5
사례토의	메르스 유행의 산발적 사례 발생에 대한 향후 대응방안 토론	1.0		AI 메뉴얼 검토	2.0	사례토의	역학조사관 제도 개선을 위한 토의	0.5
역량강화	메르스 관련 역학조사 결과 논의	2.0		에볼라 메뉴얼 검토	2.0	역량강화	EOC in Korea	0.5
				역학조사관 제도 개선을 위한 토의	5.0		대한의사협회 포스트메르스대책	2.5

Volume: 39, Article ID: e2017058
https://doi.org/10.4178/epih.e2017058

		역량강화	백일해 지침 개정 안내	2.0		언론에서 보는 의사 그리고 질병관리본부	2.5
						EIS, FETP, CSTE 등 Field Epidemiologist 제도	1.0
						지속교육 평가시험	0.5
계	6.0	계		16.0	계		15.0

역학조사관의 활동분야

역학조사관의 직무는 감염병의 예방 및 관리에 관한 법률 시행령 제 26 조 2 항에 근거하여 다음과 같다[13].

- 역학조사 계획 수립
- 역학조사 수행 및 결과 분석
- 역학조사 실시 기준 및 방법의 개발
- 역학조사 기술지도
- 역학조사 교육훈련
- 감염병에 대한 역학적인 연구

법률에 근거한 직무를 포괄하여 한국의 역학조사관들은 감염병 관리체계에서 중요한 역할을 담당하고 있으며, 다음과 같은 업무를 수행하고 있다[14].

- 감염병 집단발병(outbreak) 혹은 유행(epidemic)이 발생하였을 때 중앙정부와 시·군·구 방역당국과 연계를 갖고 유행 조사를 시행한다.
- 홍역 등의 퇴치 감염병이 사례 혹은 집단발병 형태로 발생하였을 때 사례조사와 역학조사를 시행한다.
- 예방접종 후 이상반응이 사례 혹은 집단발병 형태로 발생하였을 때 사례조사와 역학조사를 시행한다.
- 우리나라에서 토착화되어 발생하고 있지 않은 질병(예; 열대열 말라리아)이 해외에서 유입된 경우는 사례조사를 시행한다.

또한 국가 감염병 위기 상황과 관련하여 역학조사관이 큰 기여를 하고 있다[14, 15].

- 2000-2001 년 홍역 유행 조사와 예방접종 후 이상반응 역학 조사
- 2001 년 콜레라 및 anthrax hoax terror 발생 시 사례 조사
- 2003 년 중증급성호흡기증후군(SARS) 유행 시 사례 조사
- 2003 년 이후 조류 인플루엔자 유행 시 접촉자 사례 조사

Volume: 39, Article ID: e2017058
https://doi.org/10.4178/epih.e2017058

- 2009 년 신종인플루엔자 유행 시 사례 및 유행 조사
- 2015 년 중동호흡기증후군(MERS) 유행 시 사례 및 유행 조사

Table 5. 역학조사관의 주요 활동 성과

Year	Disease	Region	Investigation
2000	Malaria	National	Outbreak(32,647)
2001	Cholera	Goseong	Outbreak(18)
2003	Severe acute respiratory syndrome	National	Suspected case
2004	Hepatitis A virus	Gongju	Outbreak(105)
2005	Avian influenza virus	National	High risk group
2006	Shigellosis	Sancheong	Outbreak(198)
2007	Falciparum malaria	Busan	Confirmed case
	Human monkey pox	Seoul	Suspected case
2008	Norovirus	Churwon	Outbreak(625)
2009	Pandemic influenza A(H1N1)	National	Outbreak(>100,000)
	Hepatitis A virus	Seoul	Outbreak(33)
2010	Hepatitis A virus	Inje	Outbreak(44)
2011	Humidifier disinfectant lung injury	National	Outbreak(>100)
2012	Pertussis	Yeongam	Outbreak(154)
	Lyme disease	Hwacheon	Confirmed case
2015	Middle East Respiratory Syndrome	National	Outbreak(186)

한국의 역학조사관은 주로 공중보건의사로 구성되는데, 공중보건의사의 경우 3 년이라는 의무 복무를 마치면 개인의 자격 등을 고려하여 주로 임상 분야로 진출하고 있다. 따라서 역학조사관 교육을 통해 역학조사 혹은 감염병 관리를 충분히 할 수 있는 역량을 갖춘 뒤에 해당 업무를 마치게 되어 교육 프로그램으로서의 목적을 달성하지 못하는 실정이다. 또한 향후 공중보건의사의 수급에 제한될 수 밖에 없어 공중보건의사로 역학조사관을 구성하지 못하는 상황이 올 것으로 예측하고 있다.

역학조사관 활동으로 역학조사관 수료 이후에 역학조사관 교육 프로그램에 교육자로 참여하여, 정보 제공 및 역학조사관에 대한 사명감에 대한 서비스를 제공하고 있다.

역학조사관의 학술 성과

2010 년부터 역학조사관 교육의 내실화를 도모하기 위해 교육 프로그램의 개편이 진행되었고, 그 결과로 학술대회를 개최하여 역학조사관은 1 년에 최소한 1 편 이상의 논문을 작성하여 발표하도록 하였다. 이에 역학조사관 활동을 하며 얻어진 데이터를 정리 분석하여 교육 프로그램 2 년 차에 개최되는 학술대회에서 구연 발표를 시작하였으며, 매 해 6~8 편의 학술연구논문을 발표하고 있다. 하지만 학술활동의 대부분은 주로 중앙 역학조사관들을 중심으로 이루어지고 있으며, 시도에 파견된 역학조사관에서는 학술활동이 저조하다. 최근 2013 년과 2014 년에 발표한 연구논문은 다음과 같다(Table 6)[16, 17]

Table 6. 2013-2014년 역학조사관의 주요 학술활동 현황

연도	학술지명	제목	저자
2013	Osong public health res perspect	Spatial distribution analysis of scrub thphus in Korea	HS Jin, CS Chu, DY Han
2013	Osong public health res perspect	Epidemiological characteristics of serologically confirmed q fever cases in South Korea, 2006-2011	WS Kwak, H Chu, SD Hwang, JH Park, KJ Hwang, J Gwack, YS Choi, SK You, MY Park
2013	Osong public health res perspect	Are there spatial and temporal correlations in the incidence distribution of scrub typhus in Korea?	MS Noh, YJ Lee, CS Chu, J Gwack, SK Youn, S Huh
2013	Osong public health res perspect	Autochthonous lyme borreliosis in humans and ticks in Korea	SJ Moon, J Gwack, KJ Hwang, DH Kwon, SY Kim, YT Noh, JY Roh, EH Shin, KJ Jeong, WS Seok, SK Youn
2013	Public health weekly report	2010-2012 년 국내 렙토스피라증의 역학적 특성 분석	SJ Kim, J Gwack
2013	Infect chemother	The long and short incubation periods of plasmodium vivax malaria in korea: the characteristics and relating factors	SJ Kim, SH Kim, SN Jo, J Gwack, SK Youn, JY Jang
2013	Osong public health res perspect	Trends in the incidence of scrub typhus: the fastest growing vector-borne disease in Korea	MA Jeong, SK Youn, YK Kim, HM Lee, SJ Kim, AR Sohn
2013	Osong public health res perspect	Epidemic intelligence service officers and field epidemiology traning program in Korea	GY Kwon, SJ Moon, WS Kwak, J Gwack, CS Chu, SK Youn
2013	Public health weekly report	2001-2012 년 국내 발진열의 역학적 특성	YW Jin, J Gwack, SK Youn
2013	Public health weekly report	국내 C 형간염의 진단유병률과 위험요인	GY Kwon, HM Lee
2013	Gut and liver (in press)	Regional distribution of hepatitis C virus infection in Republic of Korea, 2007-2011	GY Kwon, HM Lee, J Gwack, SW Lee, MR Ki, SK Youn
2014	Public health weekly report	Epidemiologic characteristics of imported malaria in Korea, 2003-2012	HS kim, J Gwack, SK Youn
2014	Public health weekly report	Two imported cases of chikungunya infection in South Korea, 2013	JH Park, DH Kwon, SK Youn, JG Lee, JM Park
2014	Public health weekly report	The investigation result of a small size measles outbreak in two difference provinces in Korea, 2013	HE Eom, JH Kim

Volume: 39, Article ID: e2017058
https://doi.org/10.4178/epih.e2017058

2014	Public health weekly report	Epidemiologic and clinical characteristics of severe fever with thrombocytopenia syndrome in the Republic of Korea	JS Shin, JH Park, DH Kwon
2014	Public health weekly report	Epidemiological characteristics of legionellosis in South Korea, 2010-2013	SJ Kim, J Gwack, WC Lee, GR Bae
2014	Public health weekly report	Epidemiological characteristics of scrub typhus death in Korea, 2013	SJ Kim, HM Lee, GR Bae
2014	Japanese journal of infectious diseases	Epidemiological features and clinical manifestations of lyme borreliosis in Korea, 2005–2012	SJ Moon, YS Hong, KJ Hwang, SY Kim, JH Eom, DH Kwon, JH Park, SK Youn, AR Sohn
2014	Japanese journal of infectious diseases	An outbreak of foodborne illness caused by enteroaggregative escherichia coli in a high school, South Korea	JS Shin, SS Oh, KH Oh, JH Park, EJ Jang, GT Chung, CK Yoo, GR Bae, SH Cho
2015	Osong public health res perspect	Middle East Respiratory Syndrome Coronavirus Outbreak in the Republic of Korea, 2015	Korea Centers for Disease Control and Prevention
2015	Osong public health res perspect	Imported Melioidosis in South Korea: A Case Series with a Literature Review	SW Kim, GY Kwon, BY Kim, DH Kwon, JS Shin, GR Bae
2015	Epidemiology and Health	Strengthening epidemiologic investigation of infectious diseases in Korea: lessons from the Middle East Respiratory Syndrome outbreak	CH Lee, MR Ki
2015	Epidemiology and Health	Epidemiologic features of the first MERS outbreak in Korea: focus on Pyeongtaek St. Mary's Hospital	KM Kim, MR Ki, SI Cho, MK Sung, JK Hong, HK Cheong, JH Kim, SE Lee, CH Lee, KJ Lee, YS Park, SW Kim, BY Choi
2015	Epidemiology and Health	The first case of the 2015 Korean Middle East Respiratory Syndrome outbreak	YS Park, CH Lee, KM Kim, SW Kim, KJ Lee, JM Ahn, MR Ki
2015	Epidemiology and Health	Epidemiological investigation of the 119th confirmed Middle East Respiratory Syndrome coronavirus case with an indefinite mode of transmission during the Pyeongtaek outbreak in Korea	JH Choi, BG Yoo, SY Lee, EG Lee, MR Ki, WC Lee, JR Jung, KJ Chang
2015	Public health weekly report	Clinical Characteristics of Hand, Foot, and Mouth Disease with Complications in South Korea	BY Kim, SJ Moon, GR Bae

역학조사관의 멘토 제도

2000 년 역학조사관 교육 제도를 시작하는 시점부터 현장 교육을 지도해 주는 자문교수제도를 계획하였으나 제대로 운영되지 못하였다. 질병관리본부에 근무하는 역학조사관의 경우 근무 부서에 관련 분야의 전문가들이 다수 있어 업무를 수행하고 학습에 자문 역할을 하고 있으나, 시·도에서 근무하는 역학조사관의 경우는 지도해 줄 수 있는 현장 전문가가 부족하여 현장 교육에 미비함이 존재한다. 이에 2006 년도부터 시·도 역학조사관의 활동을 지원하는 지도교수 제도를 활성화 방안을 논의하고 구체적인 방법들이 제안되기도 하였으며, 2009 년 '역학조사관 지도교수제 도입·운영 방안 연구'를 통해 지도교수제 운영 시범사업을 진행하고 도입 시 필요한 사항들을 제안하였지만, 예산 및 제도 등이 확립되지 않아 아직까지도 미비한 실정이다. 2014 년 질병관리본부와 한국역학회 간 협정(MOU)을 체결하고, 한국역학회 소속 교수진을 추천받아

Volume: 39, Article ID: e2017058
https://doi.org/10.4178/epih.e2017058

시도에 배치된 역학조사관과 1:1 지도교수제를 운영하였으나, 그 해에만 운영되고, 그 이후로는 진전이 없었다. 운영방식은 시도에서 자율적으로 연 3 회 이상 '역학조사 업무지도 회의'를 개최하여 역학조사관이 지도교수로부터 직접 업무에 대한 지도를 받도록 하였으며, 주요 지도 사항은 시•도 역학조사관이 수행한 현장 역학조사 결과에 대한 검토 및 지도, 해당 시•도 법정감염병 발생 현황에 따른 역학조사 및 연구방향 지도, 역학조사 결과물을 바탕으로 한 학술대회 발표 및 논문화 등 학술활동 지도 등이다[4, 18].

Ⅳ. 한국의 역학조사관 교육 프로그램 평가

한국의 역학조사관 교육 프로그램의 평가는 교육 운영 평가와 교육 과정 평가로 나누어 실시되고 있다. 교육 운영 평가는 상·하반기로 질병관리본부와 교육위탁대학이 교육과정 단계별로 평가 회의를 실시하고, 1 년 과정의 교육 운영이 완료되면 종합 평가 회의를 실시하여 평가 과정 및 평가 결과를 피드백하여 차기 연도 교육 운영 계획에 반영한다. 교육과정의 평가는 교육생을 대상으로 시험을 통한 교육 내용의 지식과 실기를 평가하고, 설문지를 통해 교육과정기획, 교육운영, 매체 및 환경, 교육 성과로 나누어 교육과정 만족도를 평가하며, 교육 내용 및 강사에 대한 평가를 통해 교과목 모니터링을 실시한다[4].

역학조사관 기본교육 전후의 역량평가 결과

2015 년 역학조사관 기본교육 전·후 'FETP 역량 평가'를 실시하였다. FETP 역량평가는 CDC 에서 제시하고 있는 역량을 번역·수정하여 사용하였으며, 10 가지 영역으로 범주를 나누어 16 항목으로 평가하였다. 역량별 기술 내용에 따라 3 점 척도를 이용하여 본인이 생각하는 본인의 기술정도를 평가하였으며, 1 점은 단순 인지정도로 개념은 알고 있으나, 다른 사람의 도움 없이 업무를 수행하기 위한 기술은 불충분함을 나타내며, 2 점은 기능적 수준의 정의로 개념을 알고 있으며, 제한된 도움으로 업무를 수행할 수 있음을 나타내며, 3 점은 유능한 수준으로 다른 사람의 도움 없이 업무를 수행할 수 있으며, 다른 사람을 코치할 수 있고, 창조적인 문제해결을 할 수 있음을 나타낸다.

2015 년 역학조사관 기본교육 전·후 평가 결과 교육 전 1.43 점에서 교육 후 2.04 점으로 통계적으로 유의하게 향상되었으며($p<0.001$), 각 영역별, 역량별 기술 내용으로는 표 7 과 같다.

Table 7. FETP 프로그램 전 · 후 역량 평가 결과

영역	역량별 기술내용	N	평균		평균차	평균차의 표준편차	p 값
			전	후			
역학적 방법							
	보건프로그램 향상을 위한 연구수행에서 역학적 방법을 이용할 수 있다.	26	1.58	2.08	−0.500	0.707	0.001
	질병 유행에 대응할 수 있다.	26	1.46	2.12	−0.654	0.689	0.000
통계							
	적절한 통계방법을 이용하여 역학적 자료를 분석할 수 있다.	26	1.54	2.08	−0.538	0.761	0.001
공중보건감시							
	공중보건감시체계를 관리할 수 있다.	26	1.46	2.08	−0.615	0.637	0.000
실험 및 안전							
	역학적 활동을 지원하기 위한 실험실 자원을 활용할 수 있다.	26	1.35	1.92	−0.577	0.809	0.001
커뮤니케이션							
	공중보건 커뮤니케이션 (문서화된)을 개발할 수 있다.	26	1.38	2.08	−0.692	0.679	0.000
	구두(말하는 것)의 공중보건커뮤니케이션을 개발, 제공할 수 있다.	26	1.46	2.08	−0.615	0.637	0.000
컴퓨터 기술							
	공중보건업무와 관련한 특별한 응용을 위하여 컴퓨터를 사용할 수 있다.	26	1.62	2.04	−0.423	0.758	0.009
관리 및 리더쉽							
	현장 프로젝트를 관리할 수 있다.	26	1.35	2.04	−0.692	0.679	0.000
	직원과 자원을 관리할 수 있다.	26	1.50	2.04	−0.538	0.706	0.001
	효과적인 팀 리더 그리고 팀원이 될 수 있다.	26	1.46	2.04	−0.577	0.643	0.000
	본인의 책무를 관리할 수 있다.	26	1.62	2.23	−0.615	0.637	0.000
예방효과							
	경제성 분석을 위한 간단한 도구를 적용할 수 있다.	26	1.35	1.92	−0.577	0.703	0.000
가르침과 멘토링							
	공중보건 전문가를 훈련시킬 수 있다.	26	1.15	1.88	−0.731	0.667	0.000
	공중보건 전문가를 멘토링 할 수 있다.	26	1.19	2.00	−0.808	0.634	0.000
질병의 우선순위 결정							
	질병 또는 국가공중보건과제의 중요성을 평가하고 우선순위를 결정할 수 있다.	26	1.42	1.96	−0.538	0.706	0.001
	계	26	1.43	2.04	−0.606	0.517	0.000

역학조사관 기본교육 만족도 결과

2015 년 역학조사관 기본교육 운영에 있어서 교육생들이 평가하는 교육에 대한 만족도는 5 점 척도로 교육과정기획, 교육운영, 매체 및 환경, 교육 성과로 나누어 평가되었다. 과정 기획 영역에서는 교과목 구성이 4.5 점, 교육 내용의 난이도 4.3 점, 교육 이해 전달도 4.3 점, 교육 내용의 업무

도움이 4.3 점, 교육 방법의 적절성이 4.4 점으로 대체적으로 높은 점수를 받았다. 교육 운영 영역에서는 교육 안내 제공이 4.2 점, 원활한 진행 정도에 4.5 점이었으며, 매체·환경 영역에서는 교재 등 교육자료 활용이 4.3, 강의장 환경이 4.4 점, 식당 및 편의시설 만족도가 4.1 점이었다. 교육 과정의 전반적 만족도는 4.4 점, 전문성 및 역량 강화 4.2 점, 보건정책 및 사회발전 도움에는 4.3 점으로 대체적으로 평균점 이상으로 높은 점수의 평가를 하였다.

역학조사관 기본교육 교과목 및 강사 평가 결과

2015 년 역학조사관 기본교육 운영 결과 교과목 및 강사에 대한 평가가 수행되었다. 교과목 구성은 크게 감염병 역학 및 통계, 수인성·식품매개질환, 예방접종대상질환, 공중보건위기대응, 그 외 감염병 역학조사, 행정 관련 강의 등으로 구성되었으며, 5 점 척도로 측정되었다. 업무능력향상, 강의 전문성, 강의 기술, 강의태도를 고려한 전체 평균점수로 그 외 감염병 역학조사가 4.6 점으로 가장 높았으며, 그 다음으로 예방접종대상질환, 공중보건위기대응이 4.5 점 순이었다.

V. 문제점 및 도전점

이 장에서는 역학조사관 양성을 위한 제도개선방안, 역학조사관 교육, 훈련, 평가 프로그램 개선, 역학조사관 선발, 육성을 위한 전문교육기관 설립 운영 방안, 질병관리본부 직원 및 시도 역학조사 담당 공무원 대상 역학조사 전문인력 육성계획 수립 등에 대하여 국내·외 문헌고찰을 바탕으로 국내 예방의학 및 감염내과 등의 전문가를 대상으로 포커스 그룹 인터뷰(FGI), 포커스 그룹 인터뷰(FGI)의 의견을 바탕으로 전문가, 기존 역학조사관, 질병관리본부 직원, 시도 역학조사 담당 공무원을 대상으로 설문조사 그리고 미국 CDC 의 현장역학조사 교육훈련프로그램(FETP)/역학조사 전문요원(EIS) 담당자를 초청한 국내 전문가 및 연구진과의 워크샵을 통해 우리나라의 역학조사관의 현황과 문제점, 향후 개선 및 발전방향을 논의한 내용을 요약하였다.

역학조사관 양성을 위한 제도개선방안

현행 제도의 경우 전문성과 경험이 연결되지 않는 업무 연속성 결여가 가장 큰 문제로 인식되고 있다. 따라서 지속적, 안정적 확보가 가능하도록 정규직, 지역단위 전문역학조사관 확보 필요하다. 또한 감염병 이외의 환경성질환, 방사선, 화학 재난 등의 분야로의 확대 필요한데 이는 추후 장기적인 과제라 할 수 있겠다. 역학조사관의 권한을 강화할 경우 현행 공중보건의사제도를

병행하는 것도 대안이 될 수 있다. 역학조사관 확보를 위한 가장 기본이 되는 것으로 직업적 안정성(승진, 보수), 민간 수준의 보수, 다양한 국외 유사기관과의 협업, 역학조사관 자부심을 가질 수 있도록 하는 것이 중요하다. 역학조사관 확보를 위한 보수, 승진 및 복지 이외에도 공공의과대학 설립을 통한 안정적인 공급, 학위과정 연계, 양질의 교육과 훈련을 제공하면서 민간 수준의 보수 지급, 개방형 직위를 통하여 중견 인재의 전출입이 가능하도록, 중견 역학조사 전문 민간 교수를 영입하여 교수 직위를 부여하여 교육, 훈련을 총괄하게 하는 것, 동시에 역학조사관의 전문성을 확보하는 것 등이다. 기타의견으로 병력특례, 전문임기제에서 일정 기준 만족 시 보건복지부 및 질병관리본부 관련 직제에서 연속 근무가 가능하도록 하는 제도 시행, 기존 역학조사관(현재, 수료)에게 인센티브 제공, 장단기 해외연수 프로그램의 운영 등이 있다.

역학조사관의 사명감 고취와 비전 제시가 필요하다. 첫째, 현재의 감염병에 국한된 업무 범위를 확장하여, 만성병, 환경병 등으로의 영역 확대와 더불어 국가적 규모에서의 재난 대비와 대응 역할을 수행할 수 있는 제도적 뒷받침과 사명감의 고취가 필요하며, 둘째, 국내 우수한 인력, 기술, 경험을 바탕으로 선진국은 물론 제 3 세계 국가와의 교류를 활성화하여 역학조사관의 역량과 업무를 국내를 벗어나 확대하는 방안이 모색되어야 하며, 셋째, 전문임기제 역학조사관, 즉 질병관리본부 내의 제한된 활동을 넘어 보건복지부, 여타 중앙부처 등에서 활동할 수 있는 기회를 제공함과 동시에 일정 수준이 되었을 경우 향후 승진, 전보 등을 통해 관련 공직사회에 진출할 수 있는 기회를 제공하는 것이 중요하다.

역학조사관의 보수를 민간수준으로의 체계 개선이 필요하다. 승진적체가 갈수록 심해지고 있어 승진으로 공무원들을 동기화하는 것이 매우 제한적이다. 승진이라는 보상수단이 제한적일 경우 다른 보상수단을 통해서 구성원들을 동기화할 방안을 모색할 필요가 있으며, 이러한 경우 가장 효과적인 것이 경제적인 인센티브를 제공하는 것이라고 사료되므로, 민간수준으로의 보수 지급을 검토하여야 한다. 이외 대안으로 국내 공무원 사회에서 전문경력관 제도가 있었는데 승진의 측면에서 볼 때 향후 같은 직급의 의사 공무원이 증가하여 적체될 경우 적극 활용할 만한 제도라고 생각된다.

역학조사관의 자기계발 기회를 제공하여야한다. 근무 중 지속적으로 자기 계발을 할 수 있도록 하는 해외 연수, 국내외 학술대회 참여 등의 제도 시행이 필요하다. 군장기 인력 확보와 마찬가지로 의과대학(의학전문대학원) 학생을 대상으로 장학생 선발 제도, 군복무, 학위 제도, 예방의학 전문의 제도 등을 병행하는 복합적 접근이 필요하다.

현행 공중보건의사를 활용한 역학조사관 제도를 병행할 필요가 있다. 역학조사관은 업무의 특성상 반드시 의사가 필요한 직종이 아니지만, 역학조사는 감염병 사례이기 이전에 환자란 부문이 존재하여 보건의료에 대한 이해와 임상적 지식없이는 불가능하기 때문에 주요 활동과 의사결정은 의사를 중심으로 이루어질 수밖에 없다. 현실적으로 역학조사관에 지원하는 의사가 없다고 보건의료 전문가를 중심으로 채용할 수 없는 이유이다. 자격 요건에 일정 부문 정원을

Volume: 39, Article ID: e2017058
https://doi.org/10.4178/epih.e2017058

의사로 하여 적정 수준의 의사 역학조사관을 확보하는 일도 중요한 일이다. 현재의 과도기적 제도에서 미충족 의사 역학조사관을 보완하는 방법으로 일정 수준의 공중보건의사 역학조사관 제도를 병행 운영할 필요성이 있다고 사료된다.

역학조사관 교육, 훈련, 평가 프로그램 개선

일반적 사항으로 먼저 국내 역학조사관이 갖추어야 할 핵심역량을 설정함이 필요하다. 핵심역량에 관한 설문조사 결과, 역학조사관에게 요구할 능력과 범위, 국내 질병의 분포 등 고려하여 추후 핵심역량에 따른 교육목표 및 학습목표의 설정이 필요하다.

역학조사관 프로그램을 체계적으로 지원하기 위한 전문가 집단이나 위원회 확보가 필요하다. 전반적인 프로그램의 질 관리를 시행하고, 교육과정 중 강의나 사례연구의 개발이나 수정을 담당하고 실제로 역학조사관을 교육시킬 수 있는 인력풀의 역할을 담당할 수 있어야 한다.

질병관리본부 내 프로그램을 위한 독립된 전담인력 또는 부서 확보가 필요하다. 미국 EIS 프로그램이나 태국 FETP 의 경우 매주 세미나 시행, 주기적 역학조사관 평가, 행정적 지원 및 교육과정 관리 등이 이루어지고 있다.

교육과정에 대하여 먼저 기본교육 및 지속교육 과정은 전체적으로 5 주 내외의 과정으로 외국 FETP 프로그램보다 상대적으로 부족하다. 기본교육과정보다는 지속교육과정(2-3 일)의 확대가 필요하다. 지속교육과정을 확대하여 기본교육과정과 비슷한 강의, 사례연구, 현장실습 등으로 필요한 역량별로 진행할 필요가 있고, 기본교육과 지속교육은 필요한 핵심역량에 따른 연차별 교육이 실시되지 않았다. 핵심역량을 나누어 시기별로 교육이 필요하다. 강의의 경우 핵심역량에 따라 강의 내용을 작성하고, 핵심역량에 따라 강의시간을 배분할 필요가 있으며, 사례연구의 경우 시간을 확대하고, 지속교육에서도 적용이 필요하다. 기존의 코스워크 교육훈련(Coursework Training) 문제지의 검토 및 보완이 필요하다.

기존에 없었던 세미나 과정을 매주 진행하는 형태로 시행할 필요가 있다. 미국 EIS 프로그램이나 태국 FETP 프로그램은 매주 과제 진행 상태 발표, 북 리뷰, 추가 교육 등이 가능하다. 질병관리본부와 시도청으로 나뉘어 배치되어 있는데 화상회의(매주)나 직접 모여 세미나를 시행(매주 혹은 매달)하고 자신의 과제 발표하고 외부 및 내부 전문가를 초청하여 지도 및 피드백을 받을 수 있다.

학술대회(또는 컨퍼런스)에 다양한 분야의 전문가를 별도로 초청함이 필요하다. 발표시간 배정이 짧아 논의 없이 발표만으로 끝나기도 하는데 발표 후 심도 있는 지도와 피드백을 받도록 보완이 필요하다.

Volume: 39, Article ID: e2017058
https://doi.org/10.4178/epih.e2017058

현장 훈련과정에 있어서는 현장 관리자의 부재문제가 해결되어야 한다. 기존의 지도교수제는 적절한 지도와 피드백을 할 수 없다. 역학조사관이 배치된 기관에 현장관리자 역할을 할 수 있는 숙련된 역학자가 부족한 상황이다. 태국 FETP 자문가(advisor)의 경우 전화나 이메일로 정기적 연락, 1 년에 2 차례 해당 국가를 방문한다. 기관에 배치되어 있지 않더라도 숙련된 역학자 일부라도 확보하여 자문가(advisor) 역할을 할 수 있도록 마련하여 멘토십을 강화할 필요가 있다. 기관 내 기술적 자문가(technical advisor)나 감독관(supervisor) 형태의 지원인력이 필요한데 다양한 현장경험을 쌓기 위한 행정적 지원 및 전담인력이 필요하다. 미국의 Epi-Aid 참조가 필요하고, 배치된 기관 이외에서의 유행사례 경험이 필요한데 역학조사관 일부는 유행 역학조사 경험없이 수료하기도 한다.

평가에 있어서는 수료를 위한 요구조건 강화 고려가 필요하다. 다지역(multisite) FETP 보고서(낮은 요구조건)의 공통 부분, 유행 역학조사 완료, 감시체계와 관련한 활동, 계획된 연구에 대한 프로토콜 등이 평가되어야 한다. 역학조사관의 역량 및 과제 진척상황을 주기적으로 평가되어하는데 매 6 개월 정도로 지속 교육과정을 확대하여 병행할 수 있다(태국 FETP 의 사례). 평가를 통해 역량을 갖추고 과제를 완수할 수 있도록 지원할 수 있어야 한다. 2 년 동안 과제를 완수할 수 있도록 도움을 주는 체계 마련이 필요하다. 또한 멘토십 강화로 과제를 완수할 수 있도록 지도와 피드백이 필요하고, 역학 지원(Epi-Aid, epidemiologic assistance) 프로그램을 통하여 과제(유행 역학조사 보고서 등)를 해결할 수 있는 과정 마련이 필요하다.

역학조사관 선발, 육성을 위한 전문교육기관 설립 운영 방안

현재의 역학조사관 전문교육 운영은 교육훈련체계의 지속성 제한, 전략적 차원의 인적관리 수요조사의 미흡, 국제적인 네트워크 체계 미비, 역학조사관에 대한 단기적인 운영체계, 수료 후 공중보건 분야에 근무하기 보다는 임상 분야에 근무함에 따라 지속적인 인적자원 확보 제한 등의 문제점을 내포하고 있다.

2015 년 메르스 유행 영역별 원인을 분석한 결과 역사조사관 전문 인력의 한계와 감염병 관리정보 시스템, 감염병 교육훈련 및 대응훈련에서의 역학조사관의 수적인 부족에 다른 보건인력 자원의 조직화의 필요성과 관리의 대두성이 크게 대두되었다.

이를 해결하기 위한 역학조사관 전문교육 인력양성방안은 교육기관의 설치기준과 목적의 속성인 전문성, 체계성, 지속성이 우수성이 인정되는 가칭“역학조사관 연수원” 별도 운영 운영방안이 가장 합리적인 운영방안으로 국내·외 전문가와 현장실무자와 연구진의 의견이 대부분이었다.

그러나 이를 중·장기적으로 실천하기 위해서는 단기적으로는, 현재 질병관리본부와 건양의대에서 실시하고 있는 부분적인 국내위탁 방안을 진행하고, 이를 분석하고 운영할 수 있는 질병관리본부내의 전담인력이 구성되어 가칭 '역학조사관 연수원' 설립 위원회를 국내·외 다양한 전문가를 참여시켜 실질적인 역학조사관 전문인력을 양성하는 방법을 제안한다.

질병관리본부 직원 및 시도 역학조사 담당 공무원 대상 역학조사 전문인력 육성계획

보다 체계적인 역학조사 전문인력 육성을 위한 교육훈련제도 개발이 필요하다. 현재 교육 주관부서와 수행 기관이 교육과정별로 분절되어 있고, 역학조사 역량 강화에 특화된 교육과정이 부족한데 현재 보건복지인력개발원에서 운영하는 감염병 예방관리 및 역학조사 역량 강화를 위한 교육과정의 특성은 역학조사에 특화된 교육과정은 매우 부족하고 각 감염병별 실무능력 배양을 위한 교육과정이 주로 운영된다. 교육과정 안에 역학조사 교육내용이 일부 포함되어있을 뿐이며, 중간관리자, 과장급 이상 교육과정은 주로 일반 행정업무 역량 강화를 위한 교육내용으로 채워져 있어 중간 관리자 이상 직원들이 역학조사 업무 수행시 필요한 의사결정, 위기소통 등 역량을 강화할 수 있는 교육과정은 부재하다. 또한 교육훈련 체계와 경력관리 체계의 단절로 인해 역학조사 전문인력의 확보 및 유지의 어려움이 있는 것이 사실이다. 이를 극복할 수 있는 방안으로 단기적으로는 기존 교육훈련체계를 최대한 활용하는 방안을 제안한다. 그러나, 중장기적으로는 1 개 교육기관에서 이를 전담할 수 있도록 제도적 검토가 필요하다.
역학조사 전문인력에게 요구되는 핵심역량 개발의 추진이 필요하다. 핵심역량을 바탕으로 교육 목표와 교육 내용 선정 추진이 필요하며, 핵심역량 배양을 위한 적정한 교육방법 및 교육재료 개발 추진이 필요하고, 교육훈련 체계와 경력관리 체계와의 효과적인 통합 운영을 위한 역학조사 전문인력 양성 정책 등을 추진이 필요하다.

VI. 결론

한국의 역학조사관 제도는 제도 및 교육프로그램의 평가 방법의 개선이 필요하며, 향후 질병관리본부에서 자체적 평가를 통하여 역학조사 전문인력 양성을 위한 제도개선이 필요하다. 또한 보다 체계적인 감염병 관리 전문가 양성에 활용방안과 역학조사관 교육 프로그램의 체계적인, 단계적인 운영 기반을 구축하고 전략 개발을 위한 지속가능한 수행체계의 구축이 필요하다. 감염병에만 국한되지 않고 공중보건 전문인력 양성 및 역량 강화의 가이드라인 개정 및 전문교육기관 설립에 대한 필요성도 제기된다.

References

[1] Park NR, Jeong IS, Lee JG, Kim YT, Chun JH, Kim KS, Bae SS, Bae JM, Oh GJ, Ohrr HC, Lee KS, Lee BK, Lee HJ, Lim HS, Hwangbo Y. Evaluation of Field Epidemiology Specialist Training Program Based on the Satisfaction and the Changes of Educational Needs. *Korean J Prev Med.* 2004;37(1):80-87.

[2] Korea Centers for Disease Control and Prevention. Guideline for control and management of communicable diseases, 2015.[Korean]

[3] Han HW, Go WY, Lee HJ, Kim SS, Kim DG, Bang JH, Bae GR, Lee HR, Eun SH. Development of educational book for Korea field epidemiologist, The Report of National Institute of Health, Republic of Korea, 2003;39:27-41 [Korean]

[4] Lee MS. Final report for Korea field epidemiology training program, 2014, Konyang University, 2015.

[5] Thacker SB, Dannenberg AL, Hamilton DH. Epidemic Intelligence Service of the Centers for Disease Control and Prevention: 50 Years of Training and Service in Applied Epidemiology. Am J Epidemiol 2001;154(11)

[6]http://www.yonhapnews.co.kr/bulletin/2015/06/26/0200000000AKR20150626073000017.HTML?from=search

[7] Act for prevention and control of communicable disease (No. 13392), Republic of Korea, 2015.

[8] Dongkuk University. Development of educational course and training manual for field epidemiologist training program during 2011-2015. Osong: Korea Centers for Disease Control and Prevention; 2011

[9] Sihn KH. Research on Endemic Diseases and Japanese Colonial Rule : Focusing on the Emetine Poisoning Accident in Yeongheung and Haenam Counties in 1927. Korean J Med Hist 2009;18:173-188

[10] 朝鮮總督府, 官報 1920 年 7 月 14 日.

[11] http://www.cdc.gov/eis

[12] Regulation for operating epidemiological investigation team of Korea Centers for Disease Control and Prevention, Korea Centers for Disease Control and Prevention Sop, No. 186. 2012. 9. 19

[13] Enforcement Decree for prevention and control of communicable disease, Republic of Korea, 2015.

[14] The Catholic University of Korea. A study on efficient field epidemiologist management system in Korea, 2010.

[15] Kwon GY, Moon S, Kwak W, Gwack J, Chu C, Youn SK. Epidemic Intelligence Service Officers and Field Epidemiology Training Program in Korea. Osong Public Health Res Perspect 2013;4(4):215-221

[16] Korea Centers for Disease Control and Prevention. Epidemiological Investigation of Infectious Diseases in Korea Annual Report 2013. 2014.

[17] Korea Centers for Disease Control and Prevention. Public Health Weekly Report 2015

[18] Hanyang University. Development of Supervising System For Field Epidemiology Training Program. Seoul: Korea Centers for Disease Control and Prevention; 2009
